# Tuina (massage) therapy for diarrhea in COVID-19: A protocol for systematic review and meta-analysis

**DOI:** 10.1097/MD.0000000000021293

**Published:** 2020-07-10

**Authors:** Ke-Lin Zhou, Shuo Dong, Guo-Bing Fu, Shu-Sheng Cui, Sheng Guo

**Affiliations:** a Dongfang Hospital of Beijing University of Chinese Medicine; b School of Traditional Chinese Medicine, Beijing University of Chinese Medicine; c Beijing Gulou Hospital of Traditional Chinese Medicine, Beijing, China.

**Keywords:** complementary and alternative medicine, COVID-19, diarrhea, massage, protocol

## Abstract

**Background:**

In the beginning of December 2019, the novel coronavirus pneumonia was first detected in Wuhan, China. Its widespread infectivity and strong pathogenicity has posed a great threat to public health, seriously affecting social production and life. Accumulating evidence suggests that gastrointestinal symptoms, such as diarrhea, are common among patients with COVID-19. Tuina (massage) therapy is 1 of the widely employed complementary and alternative medicine interventions in the world. It can act on the subcutaneous muscular layer, enhance the local blood circulation and tissue metabolism of the skin, thus exert its effects on digestive systems and alleviate aversive diarrhea symptoms. This systematic review and meta-analysis will summarize the current evidence of tuina (massage) used as an intervention for diarrhea symptoms in COVID-19.

**Methods:**

We will search the following electronic databases for randomized controlled trials to evaluate the effectiveness and safety of massage therapy in treating exercise-induced fatigue: China National Knowledge Infrastructure, Wanfang and Pubmed Database, Cochrane Central Register of Controlled Trials, Cumulative Index of Nursing and Allied Health Literature, Excerpta Medica database and MEDLINE. Each database will be searched from inception to June 2020. The entire process will include study selection, data extraction, risk of bias assessment and meta-analyses.

**Results:**

This proposed study will evaluate the effectiveness and safety of massage therapy for diarrhea symptoms in COVID-19 patients. The outcomes will include the improvement of diarrhea symptoms and adverse effect.

**Conclusions:**

This proposed systematic review will evaluate the existing evidence on the effectiveness and safety of massage therapy for diarrhea symptoms in COVID-19 patients.

Dissemination and ethics: The results of this review will be disseminated through peer-reviewed publication. Because all of the data used in this systematic review and meta-analysis has been published, this review does not require ethical approval. Furthermore, all data will be analyzed anonymously during the review process.

## 1 Introduction

In the beginning of December 2019, the novel coronavirus pneumonia was first detected in Wuhan, China.^[1,2]^ Its widespread infectivity and strong pathogenicity has posed a great threat to public health, seriously affecting social production and life.^[3,4]^ The disease caused by this virus has been officially named COVID-19 (coronavirus disease 2019) by the World Health Organization (WHO).^[5]^ The coronaviruses (CoVs) belong to a large family of single-stranded, positive-sense RNA viruses that infect both humans and animals, causing several respiratory, gastrointestinal (GI), hepatic and neurologic diseases.^[6]^ The COVID-19-related disease can lead to pneumonia, Acute Respiratory Distress Syndrome (ARDS) and congestive heart failure.^[7]^ The initial research conducted on the novel-COVID-19 virus was aimed to further elucidate the epidemiological characteristics of the affected population.^[8]^ However, early studies conducted by researchers in China didn’t include digestive problems as the major health symptoms for COVID-19.^[9]^ Accumulating evidence suggests that gastrointestinal symptoms, such as diarrhea, are common among patients with COVID-19.^[10]^ Additionally, A host of patients with COVID-19 showed intestinal microbial dysbiosis with decreased probiotics, such as Lactobacillus and Bifidobacterium.^[11]^ The COVID-19 virus remains a global healthcare emergency as the number of cases and fatality continue to rise. As more information is being gathered, understanding of the virus will improve better diagnosis, prevention and treatment options for patients exposed or experiencing symptoms from the disease.^[12]^

Complementary and alternative medicine (CAM) is considered as an adjunct to treat chronic or serious diseases and to self-manage long-term health complaints.^[13]^ Traditional Chinese medicine (TCM), a main form of CAM, is an ancient and holistic approach to health and healing.^[14]^ TCM has unique theory and a long history of clinical practice. Due to its reliable efficacy and few side effects, it is widely used in the treatment of diarrhea. Tuina (massage) therapy is 1 of the widely employed CAM interventions in the world. As a useful therapy implemented on human's skin, muscles and joints, tuina (massage) has unique advantages in the field of medicine. It can act on the subcutaneous muscular layer, enhance the local blood circulation and tissue metabolism of the skin, thus exert its effects on digestive systems and alleviate aversive diarrhea symptoms. This systematic review and meta-analysis will summarize the current evidence of tuina (massage) used as an intervention for COVID-19.

## 2 Materials and methods

This systematic review protocol has been registered on PROSPERO (ID: CRD42020191952). The protocol follows the Cochrane Handbook for Systematic Reviews of Interventions and the Preferred Reporting Items for Systematic Reviews and Meta-Analysis Protocol statement guidelines.^[15]^ We will describe the changes in our full review if needed.

## 3 Inclusion criteria for study selection

### 3.1 Type of studies

This review will include clinical randomized controlled trials (RCTs) of massage therapy for diarrhea in COVID-19 patients without any language or publication status restrictions. Non-RCTs, quasi-RCTs, case series, case reports, crossover studies, uncontrolled trials, and laboratory studies will not be included.

### 3.2 Type of participants

Patients diagnosed with COVID-19 of all ages and racial groups and have diarrhea symptom will be included.

### 3.3 Type of interventions

Interventions will include any type of clinically performed massage for improvement of diarrhea in COVID-19. This will include Chinese Massage, Japanese Massage, Thai Massage, Swedish Massage, Tuina, Shiatsu, Remedial Massage, General Massage, Acupressure, Reflexology, Manual Lymphatic Drainage. Studies combined with other interventions such as acupuncture, herbal medicines, qigong and yoga will be considered for exclusion.

Control: Treatments other than massage (eg, usual or standard care, placebo, wait-list controls)

### 3.4 Type of outcome measures

#### 3.4.1 Main outcome(s)

##### 3.4.1.1 Primary outcomes

Frequency of diarrhea and quality of life. Compare the number of daily bowel movements and the texture of feces before and after treatment; compare the differences in the scores of the World Health Organization's Quality of Life Rating Scale (WHOQOL-100) before and after treatment.

##### 3.4.1.2 Secondary outcomes

Accompanying symptoms (such as myalgia, expectoration, stuffiness, runny nose, pharyngalgia, anhelation, chest distress, dyspnea, crackles, headache, nausea, vomiting, anorexia, diarrhea) disappear rate, negative COVID-19 results rate on 2 consecutive occasions (not on the same day), CT image improvement, average hospitalization time, occurrence rate of common type to severe form, clinical cure rate, and mortality.

#### 3.4.2 Additional outcome(s)

Safety measurements and adverse events.

## 4 Search methods for the identification of studies

### 4.1 Electronic searches

We will search the following electronic bibliographic databases for relevant trials:

China National Knowledge Infrastructure Database (from 1979 to present);

Wanfang Database (from 1990 to present);

Pubmed Database (from 2000 to present);

Cochrane Central Register of Controlled Trials (from 2000 to present);

Cumulative Index of Nursing and Allied Health Literature (from 1937 to present);

Excerpta Medica database (from 1947 to present);

Ovid MEDLINE ALL (Ovid Medical Literature Analysis and Retrieval System Online, from 1946 to present);

There will be no language restrictions.

### 4.2 Data collection and analysis

#### 4.2.1 Study identification

We will use EndNote X9 software to manage the records of searched electronic databases. The initial selection will involve scanning of the titles and abstracts of the retrieved studies. The full text of relevant studies will then be reviewed for study inclusion, in accordance with the inclusion criteria, by 2 authors (KLZ and SD). Potentially relevant articles will be reviewed independently by 2 authors to determine if they meet the prespecified criteria. Any disagreement between authors will be resolved by consensus with a third author. The study selection procedure will follow and be recorded in the Preferred Reporting Items for Systematic Reviews and Meta-analysis flow chart. All the evidence will be assessed by the Grading of Recommendations Assessment, Development and Evaluation.

#### 4.2.2 Data extraction and management

According to the inclusion criteria, a standard data collection form will be made before data extraction. The following data will be extracted by 2 authors (KLZ and SD):

*General information*: Research identification, publication year, the title of the study, first author;

*Study methods*: study design, sample size, randomization method, allocation concealment, blinding, incomplete report or selecting report, other sources of bias;

*Participants*: Inclusion and exclusion criteria;

*Intervention*: motion details, treatment duration, and frequency;

*Control*: Type of control methods, motion details, treatment duration, and frequency;

*Outcomes*: Included outcome measures.

#### 4.2.3 Risk of bias assessment

The risk of bias in included studies will be assessed independently by 2 reviewers (KLZ and SD) using the Cochrane Risk of Bias Tool, with any disagreements resolved by consensus or by discussion with a third reviewer. All judgments will be fully described, and the conclusions will be presented in the Risk of Bias figures and will be incorporated into the interpretation of review findings, by means of sensitivity analysis. The risk of bias of each domain will be graded as adequate, unclear, or inadequate. We intend to use the concealment of allocation grading in investigation of any heterogeneity and in sensitivity analysis. Other aspects of study quality including the extent of blinding (if appropriate), losses to follow up, non-compliance, whether the outcome assessment was standardized, and whether an intention to treat analysis was undertaken, will be presented in the risk of bias table describing the included studies and will provide a context for discussing the reliability of the results.

#### 4.2.4 Data analysis

We will use Stata Software [Computer program] (Version 15.1) to process the meta-analysis. Weighted mean difference will be used for continuous variable data, and the combined statistical effects of these 2 are combined. The *X*^2^ test will be adopted to analyze whether there is heterogeneity in each of the included research questions. *I*^2^ > 50% is a criterion for significant judgment. The fixed effect model is adopted if *I*^2^≤50%, which is considered to have homogeneity between the studies. The random effect model is adopted if *I*^2^ > 50%, which is considered to have heterogeneity among the studies. The effect size is expressed as 95% confidence interval, and *P* < .05 is considered to be statistically significant.

***Sensitivity analyses:*** heterogeneity may be due to the presence of 1 or more outlier studies with results that conflict with the rest of the studies. We will perform sensitivity analyses excluding outlier studies. In addition, we plan to perform sensitivity analysis to explore the influence of trial quality on effect estimates. The quality components of methodology include adequacy of generation of allocation sequence, concealment of allocation, and the use of intention-to-treat analysis.

***Meta-regression analyses:*** if data permits, we will perform the meta-regression analyses.

#### 4.2.5 Publication bias

If sufficient number of trials (more than 10 trials) are found, we will generate funnel plots (effect size against standard error) to investigate publication bias.

#### 4.2.6 Ethics and dissemination

The data used in this systematic review will be collected from published studies. Based on this, the study does not require ethical approval.

## Acknowledgment

The authors would like to thank all the researchers in our working group.

## Author contributions

KLZ, SD contributed on methodology and are the guarantors of the review.

KLZ, SD, and SG contributed on data search, analysis, and statistics.

**Data curation:** Ke-Lin Zhou, Shuo Dong.

**Funding acquisition:** Sheng Guo.

**Methodology:** Ke-Lin Zhou, Shuo Dong.

**Project administration:** Shuo Dong.

**Resources:** Shu-Sheng Cui, Sheng Guo.

**Software:** Ke-Lin Zhou.

**Supervision:** Guo-Bing Fu.

**Writing – original draft:** Ke-Lin Zhou, Shuo Dong.

**Writing – review & editing:** Guo-Bing Fu, Shu-Sheng Cui, Sheng Guo.
